# A Smartphone-Based System for Automated Bedside Detection of Crackle Sounds in Diffuse Interstitial Pneumonia Patients

**DOI:** 10.3390/s18113813

**Published:** 2018-11-07

**Authors:** Bersain A. Reyes, Nemecio Olvera-Montes, Sonia Charleston-Villalobos, Ramón González-Camarena, Mayra Mejía-Ávila, Tomas Aljama-Corrales

**Affiliations:** 1 Faculty of Sciences, Universidad Autónoma de San Luis Potosí, San Luis Potosi 78290, Mexico; 2 Electrical Engineering Department, Universidad Autónoma Metropolitana Iztapalapa, Mexico City 09340, Mexico; necomcontacto@gmail.com (N.O.-M.); schv@xanum.uam.mx (S.C.-V.); alja@xanum.uam.mx (T.A.-C.); 3 Health Science Department, Universidad Autónoma Metropolitana Iztapalapa, Mexico City 09340, Mexico; rgc@xanum.uam.mx; 4 National Institute of Respiratory Diseases, Mexico City 14080, Mexico; medithmejia1965@gmail.com

**Keywords:** respiratory sounds, smartphone, time-varying autoregressive model, crackles, automatic detection

## Abstract

In this work, we present a mobile health system for the automated detection of crackle sounds comprised by an acoustical sensor, a smartphone device, and a mobile application (app) implemented in Android. Although pulmonary auscultation with traditional stethoscopes had been used for decades, it has limitations for detecting discontinuous adventitious respiratory sounds (crackles) that commonly occur in respiratory diseases. The proposed app allows the physician to record, store, reproduce, and analyze respiratory sounds directly on the smartphone. Furthermore, the algorithm for crackle detection was based on a time-varying autoregressive modeling. The performance of the automated detector was analyzed using: (1) synthetic fine and coarse crackle sounds randomly inserted to the basal respiratory sounds acquired from healthy subjects with different signal to noise ratios, and (2) real bedside acquired respiratory sounds from patients with interstitial diffuse pneumonia. In simulated scenarios, for fine crackles, an accuracy ranging from 84.86% to 89.16%, a sensitivity ranging from 93.45% to 97.65%, and a specificity ranging from 99.82% to 99.84% were found. The detection of coarse crackles was found to be a more challenging task in the simulated scenarios. In the case of real data, the results show the feasibility of using the developed mobile health system in clinical no controlled environment to help the expert in evaluating the pulmonary state of a subject.

## 1. Introduction

Chronic respiratory diseases (CRDs) are among the principal causes of mortality and morbidity around the world, according to the World Health Organization [1]. The first approach employed in the diagnosis of pulmonary diseases is the clinical examination of the pulmonary function that includes clinical history and auscultation of the lungs with the stethoscope. During the auscultation procedure, adventitious lung sounds added on the breath or base lung sounds are a common finding. Although the stethoscope remains the most widely used instrument in clinical medicine and still guides diagnosis when other pulmonary function tests are not available, the auscultation by the stethoscope has several limitations, e.g., it is a subjective process that depends on the ability and expertise of the physician [2], it is limited by human audition [3], the stethoscope may be more adequate for cardiac auscultation [4], and the lung sounds are not permanently recorded for further analysis.

Nowadays, it is recognized that respiratory sounds (RS) make it possible to obtain information about the respiratory health of a subject in a non-invasive fashion, e.g., the characteristics of RS differ between different pulmonary disorders, reflecting different pathophysiologies and severity levels [5]. To overcome the limitations of the auscultation, the use of Computerized Respiratory Sound Analysis (CORSA) systems has been proposed [6]. The use of CORSA systems also helped to arrive at a classification of RS, and nowadays they are understood as those sounds produced while breathing and classified according to their characteristics as breath sounds (basal sounds) or adventitious sounds (added or superimposed sounds) [7]. In turn, adventitious sounds are classified according to their duration as continuous, e.g., wheezes, and discontinuous or crackle sounds. In particular, crackle sounds are traditionally classified according to their temporal characteristics as fine (short duration) or coarse (long duration) crackles [3]. Among the adventitious sounds heard during auscultation, crackle sounds are a usual finding in patients with hypersensitive pneumonia (HP), chronic obstructive pulmonary disease, bronchiectasis, and other diffuse interstitial pneumonia diseases [8]. For audiovisual detection of crackles, the use of CORSA systems, via the method of time-expanded waveform analysis (TEWA)**,** is an improvement when compared to the auscultation [9]. However, the detection of crackle sounds by TEWA is a challenging task due to crackle’s temporal and spectral characteristics, i.e., their transient and short lasting behavior (<20 ms), broad frequency content with their main power ranging from 100 Hz to more than 1 kHz, time-varying spectra that decreases over time [10], temporal overlapping of crackle waveforms and low signal-to-noise ratios along the respiratory phases. Accordingly, several methods have been proposed to automatically detect crackle sounds including, non-linear digital filtering [11], fuzzy logic-based filters [12], wavelet-based filters [13], fractal dimension analysis [14], empirical mode decomposition [15], independent component analysis [16], time-varying autoregressive modeling [17], and time-frequency analysis [18,19]. Unfortunately, these algorithms were designed and implemented in traditional CORSA systems which have been mainly conceived to specialized research and clinical settings. Furthermore, these systems may not be easily translated to the primary point-of-care settings because their limited mobility, high costs and low integration between acquisition and analysis stages, i.e., processing of the information is usually performed offline by means of commercial and general purposes numerical computer software.

As an alternative to traditional CORSA systems, mobile health CORSA (mHealth CORSA, mCORSA) systems have been proposed in the field of respiratory sounds. According to the WHO, mHealth is understood as the use of mobile and wireless technologies to support the achievement of health objectives and it has the potential to transform the ways of health delivery around the world [20]. Nowadays, mobile devices like smartphones and tablets have characteristics like being ubiquitous, equipped with multiple cost-effective sensors, continuously updated in software and upgraded in hardware, embedded multimedia capabilities, and tactile interaction with the user, that give them advantages over other architectures in terms of implementation an integration with other health monitoring systems [21]. Around one fifth of the world population owns a smartphone and their market penetration continues to rise. In addition, both patients and healthcare professionals have reported multiple reasons for adopting mHealth systems, e.g., ability to obtain information not easily granted or impossible to get without these applications, to take a greater control of their healthcare, and to reduce healthcare costs [22]. In the field of respiratory sounds, several efforts have been done to develop mCORSA systems including mobile applications (apps). For example, smartphone-based systems have been employed to develop an asthma monitoring system using wireless communications [23], to record respiratory sounds for snoring and sleep apnea severity [24], to record respiratory sounds for snoring detection [24], to study the characteristics of RS and to extract respiration-related information from them like respiratory frequency [25] and tidal volume [26], to detect respiratory symptoms like sneeze and cough [27], to record wheezes in pediatric populations [28], to record respiratory sounds with snores for classification purposes [29], and to record respiratory sounds for analysis of obstruction sites in patients with obstructive sleep apnea [30]. Hence, smartphone-based systems have been particularly used to record respiratory sounds and analyze continuous adventitious sounds, i.e., wheezes and snores, that result helpful in the study of asthma and sleep apnea. An extensive review of mobile apps developed particularly for snoring can be found in [31]. Efforts regarding crackle analysis and detection have been more limited. A smartphone-based system with an app using a time-frequency analysis for detection of wheezes have been reported, and although there was mention to crackle sounds, just their time-frequency representation was presented, leaving the user the task to manually detect them and count them [32]. A similar scenario was reported where a smartphone system was developed for automatic classification of adventitious sounds. Results were presented in terms of wheezes and although there was mention about the presence of crackle sounds in the recordings, details about their detection or the algorithm employed was not provided [33]. To the best of our knowledge, a smartphone-based system designed with capabilities for automated detection of respiratory crackle sounds directly in the mobile device is still missing.

The presence of crackle sounds is considered to reflect a pathological process in pulmonary airways and tissue [3]. In addition, the timing of crackle sounds during the respiratory cycle has been found to reflect different pulmonary disorders, e.g., presence of crackles during the initial/final portion of the inspiration have been associated with restrictive pulmonary diseases/severe airway obstruction [34] while expiratory coarse crackles are found to be less frequent than inspiratory crackles but they specially occur in chronic airway obstructions [35]. However, crackles could be missed by traditional auscultation due to their very short duration and often low intensity for which the human audition has limitations. To overcome the former limitations, in this study a smartphone-based system for recording and processing of respiratory sounds including an algorithm for the automated detection of crackles is introduced. The detection algorithm was implemented in an Android app and is based on modeling the respiratory sounds as output of a time-varying autoregressive (TVAR) equation where the TVAR coefficients are estimated via the recursive least squares (RLS) algorithm. In addition to the counting of crackles, their timing is also provided by the algorithm and the results summarized in terms of their occurrence in the breathing cycle. The performance of the algorithm was tested using both simulated scenarios inserting synthetic crackles in acquired basal sounds from healthy subjects and data acquired from patients with respiratory diseases.

## 2. Material and Methods

### 2.1. Healthy Subjects and Diffuse Interstitial Pneumonia (DIP) Patients

For this study, two sets of respiratory sounds recordings were employed: (1) from ten (*N* = 10) healthy subjects to test the performance of the crackle detector in controlled scenarios with inserted crackles, and (2) from nine (*N* = 9) patients with DIP, to test the performance of the crackle detector with real data found in the clinical environment. Both sets of recordings were performed at the National Institute of Respiratory Diseases (INER) in Mexico City. All the subjects gave their signed informed consent for participation in the study approved by the Ethics Committee of INER (Project ID number: C19-12, Approval date: 11 January 2017). Investigations were carried out following the rules of the Declaration of Helsinki of 1975, revised in 2008.

#### 2.1.1. Acquisition from Healthy Subjects

Respiratory sounds from ten (*N* = 10) healthy, non-smoking volunteers with an age 24.3 ± 1.5 years, weight 77.8 ± 11.00 kg and height 174.8 ± 7.8 cm, were acquired. All volunteers were residents of Mexico City located at 2240 m above sea level. Exclusion criteria included individuals with previous pneumothorax, those with chronic respiratory illnesses such as asthma, and anyone who was currently ill, e.g., common cold or upper respiratory infection. Respiratory sounds were acquired at posterior left basal lung locations using acoustical sensors consisting of subminiature electret microphones coupled in air bells and having a flat frequency response in the range of 50–3000 Hz. In addition to the respiratory sounds, airflow was acquired with a previously calibrated pneumotachometer (Fleisch, KS, USA) and located in front of the volunteers for visual feedback purposes. During acquisition, volunteers were standing still and were asked to breath at a maximum airflow of 1.5 L/s for at least five respiratory cycles. Sounds and airflow data were simultaneously acquired at a sampling frequency *fs* = 10 kHz and digitized with a 12-bit A/D card. Data was stored in a binary file for their further analysis using Matlab (The Mathworks Inc., Natick, MA, USA) and using the developed smartphone app.

#### 2.1.2. Acquisition from DIP Patients

Recordings from nine (*N* = 9) patients diagnosed with DIP, with ages ranging from 43 to 65 years (65.11 ± 12.47 years, mean ± std), were acquired using the developed smartphone-based system described in the next section. Three recordings were acquired from each patient and they were performed at the bedside where flow or volume measurement was not available. Hence, to perform the respiratory maneuver, patients followed a volume-like signal displayed in the developed smartphone app as a visual feedback, in conjunction with a guidance of the physician. In this way, this respiratory maneuver allowed to have a reference signal useful to determine the location of crackle sounds in respiratory phases, i.e., inspiration or expiration. Respiratory sounds were recorded at pulmonary zones indicated by the physician after he performed a pulmonary auscultation with a mechanical stethoscope to find the presence of crackle sounds. In the same way as healthy subjects, all the patients gave their signed informed consent for participation in the study approved by the Ethics Committee of INER (Project ID number: C19-12, Approval date: 11 January 2017) in accordance to the rules of the Declaration of Helsinki of 1975, revised in 2008.

### 2.2. Smartphone-Based System for Automatic Crackle Detection

#### 2.2.1. Hardware

**Figure 1:**
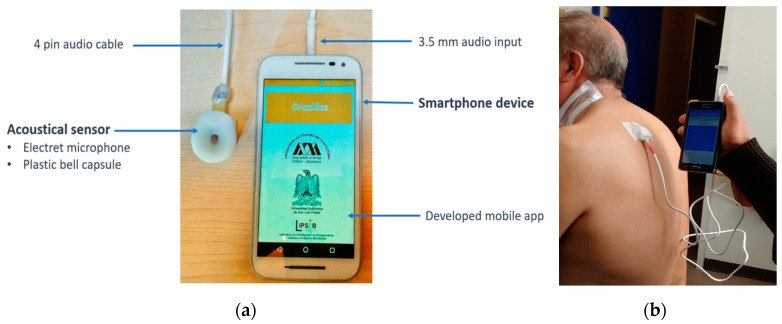
(**a**) Smartphone-based system developed for the automatic detection of respiratory crackle sounds for displaying, processing, and provide crackle detection results, (**b**) Acquisition at bedside of a patient diagnosed with diffuse interstitial pneumonia.

The proposed mHealth system, shown in Figure 1, consists basically of two hardware components: (1) an electret subminiature microphone (BT-2159000, Knowles Electronics, Itasca, IL, USA) encapsulated in a plastic bell designed for adequate acquisition of respiratory sounds and connected to the 3.5 mm audio input of the smartphone [17], and (2) a smartphone device containing the developed mobile app governing sounds acquisition, display and processing. The developed app was mainly tested in two Android smartphone devices of low and medium performance classes, the Galaxy S4 (Samsung Group, Seoul, Korea)**,** with 1.6 GHz quad-core processor, 2 GB RAM and running Android v5.0.1—Lollipop, and the Moto G Turbo Edition (Motorola, Chicago, IL, USA), with 1.5 GHz octa-core processor, 2 GB RAM and running Android v6.0—Marshmallow. It is worth mentioning that both smartphone devices provide reliable digitalization requirements as recommended in guidelines for respiratory sound acquisition [36]. Respiratory sound recording using the developed system is illustrated in Figure 1 for a DIP patient.

#### 2.2.2. Mobile Application (App)

In addition of being the world’s most popular mobile operating system (OS) and given the experience of our research group in developing smartphone apps, the app for this work was developed for the Android mobile OS (Google Inc., Mountain View, CA, USA). The mobile app was developed with the official Integrated Development Environment (IDE) Android Studio (Google Inc.) using XML (W3C, Cambridge, MA, USA) and Java (Oracle Corp., Redwood Shores, CA, USA) programming languages on a personal computer running Windows 10 OS with Intel i7-6500U processor (Intel Corp., Santa Clara, CA, USA) with 4 MB Cache and up to 3.10 GHz processing frequency, 16 GB of RAM, and NVIDIA GTX 950 graphics card (NVIDIA Corp., Santa Clara, CA, USA). Android Studio allowed testing the developed app using emulated devices in addition to finally test the app on both the Galaxy S4 and Moto G physical smartphones. The mobile app was designed to satisfy functionality requirements stated by the pneumologist collaborators. To complement the automatic crackle detection algorithm, the mobile app allows the acquisition and storage of respiratory sounds from new or existing patients as well as audiovisual display of the sounds.

#### 2.2.3. App Activities and Graphical User Interface

**Figure 2:**
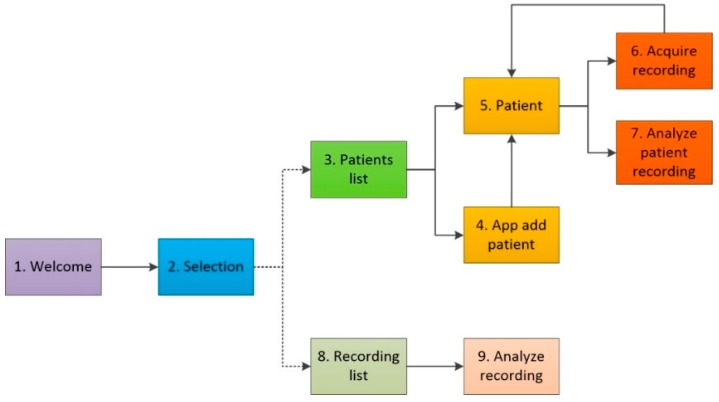
Mobile app flowchart for the automated detection of respiratory crackles. In the flowchart each block represents an activity that was implemented in the app.

To achieve the abovementioned functionalities, a set of app activities were designed and implemented in the Graphical User Interface of the app and organized as shown in Figure 2. It is worth mentioning that the main libraries employed in the development of the mobile app were Audio Record to record sound signals, Media Player to reproduce the recorded sound signals [37] and MPAndroid Chart to display the recorded sound signals [38]. A diversity of classes and methods were implemented in Java to perform all the signal processing stages like digital filtering employing finite impulse response filter designed with the windows method and the Fast Fourier Transform algorithm. A description of the task performed for each activity is as follows:

1. *Welcome activity* (*WA*). Two seconds splash-screen that contains the application name and affiliation logos of the developers. In addition, *WA* asks the user for permissions for memory reading/writing, recording, and playing of audio files. Otherwise, the app does not allow to continue. The first time the app is opened, and the permissions conceded, a folder called *Crackles* is created to store the patient’s information and their recordings obtained with the app. Additionally, a folder called *AnotherSignals* allows to store signals obtained with other systems and transferred to the smartphone for audiovisual display and analysis by the app. Finally, a validation of the sampling frequency, equal to 10 kHz, is performed. If not supported, the app does not allow to continue.
2. *Selection activity* (*SA*). *SA* allows the user to choose between two data visualization options: (1) list of registered patients, and (2) list with all existing recordings. SA also contains menu buttons to visualize the app developing credits.
3. *Patients list activity* (*PLA*). *PLA* displays a list with all the registered patients in the app so that the user has the option to choose an existing patient from the list or to add a new patient via a floating action button (FAB).
4. *App add patient activity* (*APA*). This activity allows to add a new patient by introducing his/her surname, given name, gender and age. Alphabetic or numeric keyboards are displayed depending the case. The app does not allow to add a new patient until all required fields have been filled and validates the provided information preventing duplicates.
5. *Patient activity* (*PA*). The *PA* app displays patient information and a list with his/her existing sound recordings so that one can be selected to analyze it, or the user can opt to acquire a new recording.
6. *Acquire recording activity* (*ARA*). *ARA* allows the acquisition of a new respiratory sound recording at a specific auscultation point by following a respiratory maneuver designed for this study.
7. *Analyze patient recording activity* (*APRA*). This activity allows to analyze respiratory sounds to automatically detect crackles. After the analysis, the time locations of the detected crackles are stored in a text file and *APRA* displays the obtained results in terms of:
  o A figure displaying the acquisition location (auscultation point)
  o A table with a summary of the crackles counting, as well as the total amount of crackles detected
  o A graph with the whole respiratory sound recording and location of the detected
  o crackles. This graph cannot be manipulated
  o A graph with a segment of the respiratory sound with tactile manipulation capabilities for temporal zoom and scroll. In addition, the user can play the audio.
8. *Recording list activity* (*RLA*)*. RLA* performs as a file explorer displaying a list of folders and files contained in the folder *OtherSignals* so that the user can analyze it.
9. *Analyze signal activity* (*ASA*)*.* It allows to detect crackle sounds in the selected recording manually added by the user, i.e., in respiratory signals acquired with other systems. The app allows the user to perform pre-processing (filtering and normalization). After the signal analysis finishes, detection results are presented in terms of the number of detected crackles in conjunction with the two graphs of respiratory sounds explained above.

**Figure 3:**
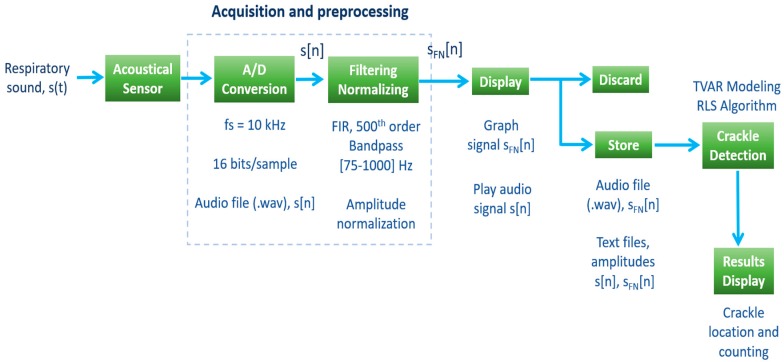
Block diagram of the acquisition, processing and display of results from respiratory sounds with the developed mobile app.

A block diagram summarizing the acquisition and processing of the respiratory sounds performed in the app is shown in Figure 3. In the next subsections, details about the acquisition and preprocessing of respiratory sounds as well as the crackle detection stage are provided.

#### 2.2.4. Acquisition and Pre-Processing of Respiratory Sounds

Respiratory sound signals s(t) were digitized using the internal A/D converter of the smartphone devices at *fs* = 10 kHz using 16 bits-per-sample. The app temporary stores an audio file in .wav format to be played by the physician before choosing to store or discarded it and acquired a new one. The smartphone-acquired sounds are digital filtered in the mobile app using a 500th order finite impulse response (FIR) bandpass filter between 75 and 1000 Hz to reduce the possible presence of heart sounds and other muscular noises. Filtered sound signals are normalized in amplitude in the range [−1, 1] to account for different amplitude variations between recordings. Both raw acquired signal s[n] and its filtered and normalized version $s_{FN}\left[n\right]$ can be discarded by the user after their audiovisual display so that another acquisition can be performed. If decided, both s[n] and $s_{FN}\left[n\right]$ time series can be stored in the mobile app in text and audio files for further visualization and analysis in terms of crackle detection directly in the smartphone device.

#### 2.2.5. Selection of Auscultation Points and Respiratory Maneuver

**Figure 4:**
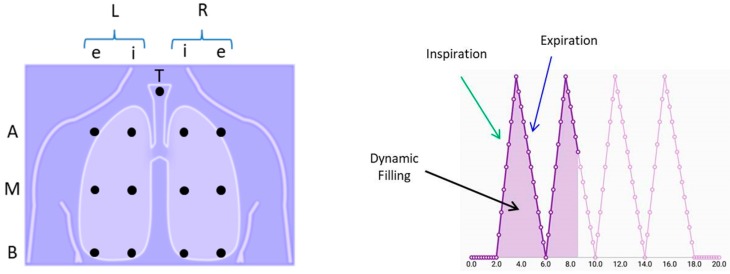
Auxiliary activities for the acquisition of respiratory sounds in the developed app. **Left**: In the mobile screen an array of possible acquisition locations appears to associate a name to the acquired sound. **Right**: a second screen is shown by the app where the respiratory maneuver with dynamic filling provide a visual guidance to the physician and patient about the inspiration and expiration timing.

Before starting the acquisition of respiratory sounds, the *Acquire recording activity* allows the user to graphically select an auscultation point from a schematic array of locations proposed by our research group to cover the thoracic area as shown in Figure 4. The nomenclature for the 3 × 2 array consists of:

- Lateral side of the thorax. L: left, R: right
- Thoracic row level. A: apical, M: medial, B: basal
- Hemithorax line level. e: exterior, i: interior

Accordingly, a sound recorded over the basal region of the right hemithorax at the exterior line would be denoted as LBe by the app. Because our research group and collaborators are interested in analyzing also tracheal sounds, an auscultation point on the trachea (T) is also available for tactile selection in the array. Similar auscultation points have been proposed by other research groups to facilitate the registration of recording locations [33]. In the case of the array proposed in this work, it corresponds to a simpler version of the previously introduced by our research group for multichannel acquisition and processing of respiratory sounds [39].

After selecting an auscultation point, the *Acquire recording activity* allows the user to control the sound recording. Because the low availability of a spirometer, or another respiration activity recording system, in primary care settings or it is commonly underutilized [40,41], a respiratory maneuver was designed in order to provide a temporal reference from which information about the respiratory phases, i.e., inspiration (I) and expiration (E), can be obtained, see Figure 4 right side. Accordingly, a respiratory maneuver consisting of 2 s of initial apnea, four respiratory cycles with a duration of 4 s each with I:E ratio of 2:3, and 2 s of final apnea, was designed. A visual feedback was implemented in the app activity so that the area under the maneuver curve is filled to aware the physician and the patient about when to inspire and expire. At the end of the recording, the physician can replay the sound and decide between discard or stored it to analyze it with the crackle detection algorithm implemented in the app.

#### 2.2.6. Time-Varying Autoregressive Algorithm for Automated Detection of Crackles

The rationale behind the algorithm implemented in the app for the automated detection of crackles was that crackles are nonstationary events that provoke abrupt changes in the coefficients of a time-varying autoregressive (TVAR) model in comparison to the basal respiratory sound [17]. For a discrete stochastic signal $s_{FN}\left[n\right]$, the filtered and normalized version of acquired respiratory sound signal, its TVAR model of order M is given by:

(1) $$\;s_{FN}\left[n\right]=-\overset{M}{\underset{k=1}{\sum }}a_{k}\left[n\right]s_{FN}\left[n-k\right]+v\left[n\right]\;$$

where the set of ${\left\{a_{k}\left[n\right]\right\}}_{k=1,\cdots ,M}$ are the TVAR coefficients at time *n*, $s_{FN}\left[n-k\right]$ are past samples of $s_{FN}\left[n\right]$ and, and v[n] is a white noise process [42]. The developed mobile app estimates the TVAR coefficients at each time instant using the recursive least squares algorithm (RLS) with constant forgetting factor, λ, to controls the influence of prior information while minimizing a cost function ξ[n] defined by

(2) $$\;\xi [n]=\overset{n}{\underset{i=1}{\sum }}\lambda^{n-i}{\left|e\left[i\right]\right|}^{2}\;$$

where e[n] refers to the error of the adaptive filter. Details about RLS algorithm can be found elsewhere [42].

**Figure 5:**
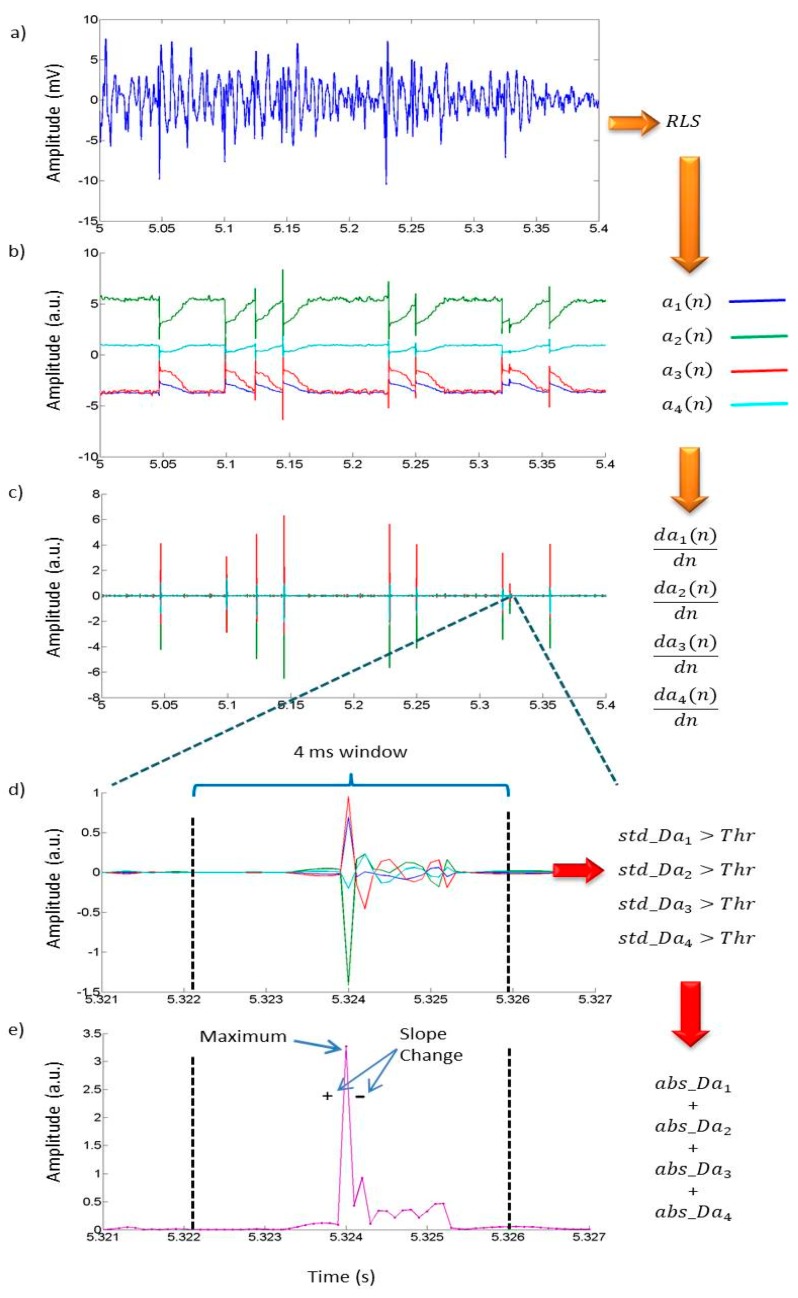
Schematic illustration of the processing steps implemented in the mobile app for the automated detection of respiratory crackles. (**a**) The sound signal *s_FN_*[*n*] is modeled using a 4th order TVAR model. (**b**) RLS-TVAR coefficients and their abrupt changes by the presence of crackles around 5.05 s, 5.1 s, etc., (**c**) Derivates of the coefficients time series in (**b**) where the abrupt changes are emphasized. (**d**) Segmenting of each coefficients time series derivates (TSD) by sliding 4 ms window where a threshold value is employed to determine presence of crackles. (**e**) If presence of crackles is detected inside a window for all the derivates then add the absolute values of the TSD and the maximum is found. The time location of the maximum is regarded as the starting point of the detected crackle sound.

The RLS algorithm implemented in the *Analyze Patient Recording activity* employs a 4th order TVAR model (M = 4), and a forgetting factor close to the unity, *λ* = 0.97. This selection was previously found to be adequate for crackle detection in a previous effort by our research group, which in turn was based on Akaike’s criterion [17]. Once the TVAR coefficients of the signal $s_{FN}\left[n\right]$ are computed, their abrupt changes are detected via the local maxima in the slopes of the sum of the absolute value of the TVAR derivatives by employing a threshold value equal to thr = 0.024 and a 4 ms moving window. The threshold parameter was set using a grid search approach as described in the results section. The overall implemented algorithm for automatic detection of respiratory crackle sounds is schematic illustrated in Figure 5 and consists of the steps described in Table 1.

### 2.3. Performance Evaluation of the Crackle Detection Algorithm

The performance of the crackle detector implemented in the mobile app was tested in two different sets of data. First, controlled scenarios with synthetic crackle waveform were generated and randomly inserted in the acquired respiratory sounds from healthy volunteers. Second, real acquired respiratory sounds were recorded at the bedside of patients with diffuse interstitial pneumonia.

#### 2.3.1. Simulated Scenarios of Respiratory Sounds with Crackles

Synthetic fine and coarse crackles were generated and inserted at random locations in the acquired respiratory sounds from ten healthy volunteers in the following six different scenarios intended to reflect conditions found in clinical practice, see Table 2.

**Figure 6:**
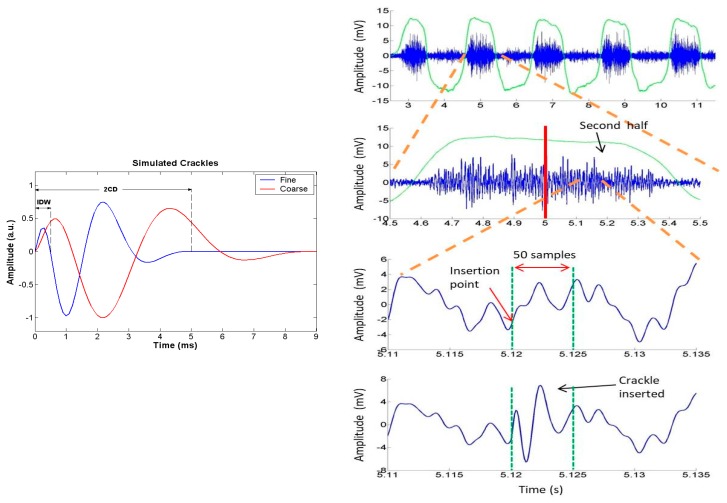
Simulated scenarios with synthetic crackle sounds. **Left**: Waveform of simulated fine and coarse crackles. **Right**: Insertion of a synthetic fine crackle at a random location using a gain factor equal to 3.5 times the variance of the basal sound in the insertion window.

Synthetic crackles were generated by amplitude modulating an oscillatory signal whose frequency content decreases over time according to the following equation with normalized duration:

(3) $$\;x\left(t\right)=sin^{2}\left(\pi \sqrt{t}\right)\;\sin\left(4\pi t^{\frac{\log\left(0.25\right)}{\log\left(T_{0}\right)}}\right),\;\;0\leq t\leq 1\;$$

where $T_{0}$ refers to the initial deflection width (IDW) and the two cycles duration (2 CD) corresponds to the crackle duration. The IDW parameter was set to 0.5 and 1.2 ms, and the 2 CD parameter was set 5 and 9 ms, for the generated fine and coarse crackles, respectively. For each scenario, the synthetic crackles were randomly added to base respiratory sounds with three different gain factors equal to 1.5, 2.5 and 3.5 times the variance of the basal sound in the corresponding insertion window to simulate different signal-to-noise-ratios (SNR). Figure 6 shows the generated fine and coarse crackles and illustrates the insertion procedure for a fine crackle at the end of an inspiration.

#### 2.3.2. Performance Indices

The simulated scenarios in Table 2 allow to know the exact amount and location of inserted crackles. Accordingly, the indices of accuracy (Acc), sensitivity (Sen), and specificity (Spe) were employed to quantify the performance of the implemented crackle detector according to:

(4) $$\begin{array}{c} \;Acc=\frac{TP}{TP+FN+FP}\\ \;Sen=\;\frac{TP}{TP+FN}\\ \;Spe=\;\frac{TN}{TN+FP}\; \end{array}$$

where *TP* are the true positives (correct detection), *FN* are the false negatives (missing detections), *FP* are the false positives (extra detections), and *TN* (correct no-detection). A detection was counted as correct if the detection point was at a distance less or equal than 3 samples from the real insertion point. In addition, the absolute time difference $\left|\Delta t_{loc}\right|$ between the true location of the inserted crackle and the detected location with the implemented app was quantified.

#### 2.3.3. Respiratory Sounds from DIP Patients

Regarding the acquired respiratory sounds from patients, their labeling, i.e., manual location and counting of crackle sounds across the recordings, was performed by pneumologists using an audiovisual GUI developed in Matlab with capabilities to perform TEWA analysis, i.e., time expansion of the signals. It is worth mentioning that labeling of respiratory sounds using TEWA criteria is a cumbersome and time demanding procedure considering that recordings have a much longer duration (around 20 s) compared to the events of interest (around 10 ms). As a mean to aid the manual labeling, audio reproduction and time-frequency analysis of the respiratory sounds were also provided to the GUI. To this end, physicians were asked to analyze one respiratory cycle of each acquired recording. In contrast to the simulated scenarios, the exact beginning location and amount of crackles is not known or cannot be obtained without subjectivity. Accordingly, a double-blind approach was employed to test the performance of the algorithm by comparing the amount of crackles obtained with the mobile app with the amount and location of crackles provided by the physician.

## 3. Results

### 3.1. Mobile Application (App)

**Figure 7:**
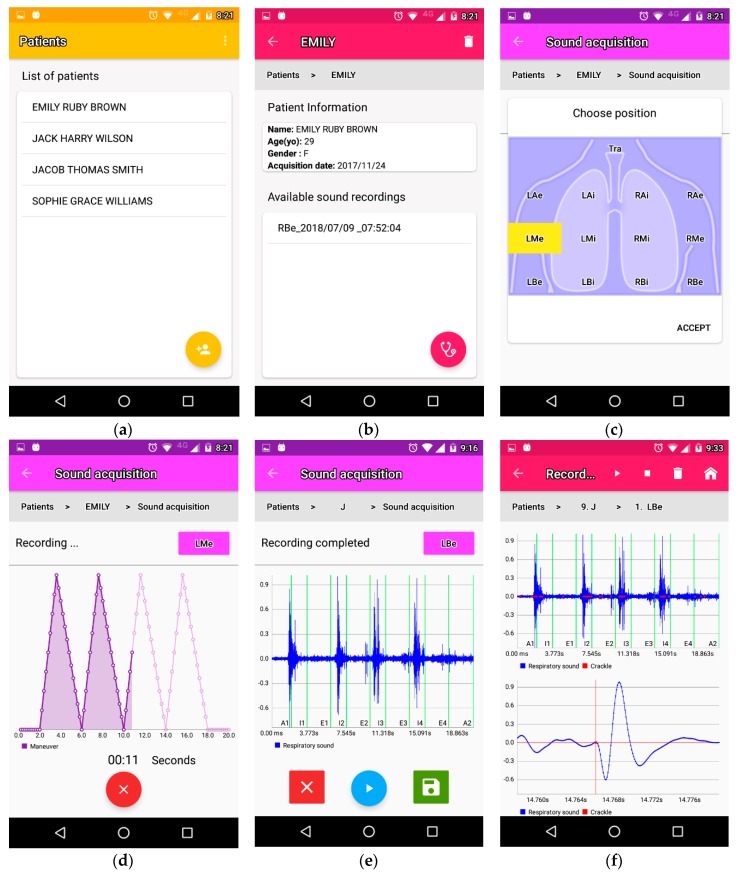
Different activities of the mobile app. (**a**) Patient list activity. (**b**) Patient activity. (**c**) Selection of auscultation point activity. (**d**) Sound recording activity. The area under the curve fills during the acquisition to provide a visual aid. (**e**) Activity to replay, discard or save the recording. (**f**) Analyze patient activity. The upper graph displays the whole recorded respiratory sound and the detected locations of crackles. This graph is fixed. The bottom graph displays a segment of the signal selected by the user so that it can be zoomed, scrolled and tactile select a data point to know its exact time location on the screen.

The implemented Android app requires 10 MB of space to be installed on smartphone devices. Screenshots of the implemented app are shown in Figure 7. The *Patient List* activity that displays the list with patients already registered is shown in Figure 7a. A FAB also allows the user to register a new patient. Figure 7b shows the *Patient activity* which displays the entered information of a patient. A list with the sounds already recorded for that patient is displayed in the inferior portion of the app so that the user can select one to analyze it or can use a FAB to acquire a new respiratory sound recording. Figure 7c,d shows the activities concerning the sound acquisition. The selection of the auscultation point is performed by touching a location in the graphical array (Figure 7c) and once selected, the sound acquisition activity is started so that the user can start the recording while simultaneously receiving visual feedback about the respiratory maneuver to performed by the patient (Figure 7d). The user can replay the acquired sound to analyze its quality and decide if discard it or store it for further analysis in the app (Figure 7e). Figure 7d shows the activity in charge of displaying the analysis results in terms of crackle detection. The location of the crackles is displayed as red markers on top of the respiratory sound. The user can manipulate a graph in the lower portion of the activity in terms of scrolling and zooming. In the presented example, an interval containing one crackle sound detected from a recording from a patient with respiratory disease is displayed in the zoomed version of the signal. As can be seen, the detected crackle sound resembles the morphology reported in the classical literature [3,9]. Regarding the performance of the developed app, the average computation time employed to process the recorded signals was around 3.5 min and was distributed as follows, 195 s for mean removal and digital filtering, 0.8 s for signal normalization, 17.9 s for signal display, and 14.1 s for automated crackle detection. In addition, it was found that the mobile app employed around 2% of the battery when 5 consecutive data acquisitions and processing, including crackle detection, were continuously performed and starting from a full charge in a 2740 mAh device, i.e., it consumed around 55 mAh in a commonly available smartphone device. Finally, it was found that, on average, only 20 MB of RAM were employed by the mobile app to run on the smartphone device.

### 3.2. Estimation of TVAR Coefficients in the App

**Figure 8:**
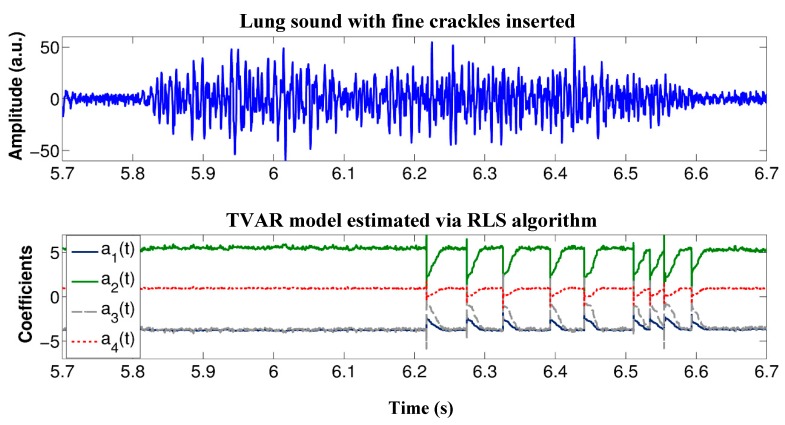
Example of RLS-TVAR coefficients estimates using the implemented mobile app. **Top**: Time waveform of an inspiratory sound with inserted fine crackles at the end portion. **Bottom**: Time course of the RLS-TVAR coefficients estimated in the app.

**Figure 9:**
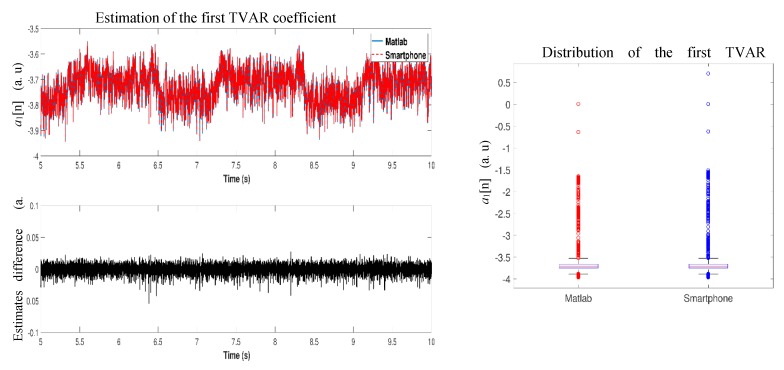
Comparison of TVAR coefficients estimated using the implemented RLS algorithms in the mobile app and Matlab. **Left**: Example of estimates and difference between the estimates in both systems. **Right**: Distribution of the TVAR coefficient estimated in both systems.

The implemented algorithm for automated detection of crackle sounds relies on the estimation of the TVAR coefficients via the RLS algorithm with fixed forgetting factor. An example of the TVAR coefficients estimated using the implemented Android app is displayed in Figure 8 for a simulated scenario with fine crackles inserted at the end the inspiratory phase. The $a_{0}$ coefficient is not shown as its constant valued equals to 1. In Figure 8 is possible to notice the presence of simulated crackles in the respiratory sound by looking at the transients in the TVAR coefficients time series. The implementation of the RLS algorithm was tested by comparing the TVAR coefficients estimation results with those provided by a Matlab implementation using one recording. The estimated coefficients used to test the statistical significance were obtained completely in each system; i.e., after performing digital filtering; normalization and applying the RLS algorithm in a personal computer and in the app using the developed system to the same recording. No statistically significant differences were found between the two sets of estimates as analyzed by a Wilcoxon signed-rank test for any of the TVAR coefficients; *p* > 0.99 for $a_{1}\left(t\right)$ to $a_{4}\left(t\right)$. Figure 9 shows the distribution for a TVAR coefficient using a box plot as well as the amplitude difference versus time between its estimation using Matlab and the implemented app.

### 3.3. Performance of the App for the Detection of Crackle Sounds

**Figure 10:**
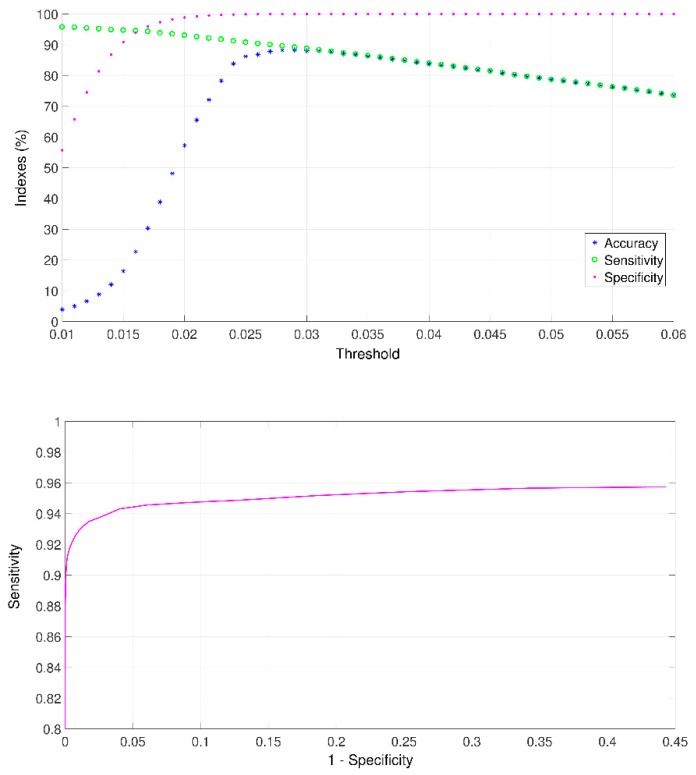
Performance indices of the developed automated crackle detector versus threshold value. **Top**: accuracy, sensibility and specificity indices. **Bottom**: Receiver operating characteristic (ROC) curve of the developed automated crackle detector. Results are for all simulated scenarios in Table 2.

A relevant parameter for the mobile app is the selection of the threshold value. This selection was performed by varying its value from 0.010 to 0.060 and obtaining the performance indices introduced in previous section for all the simulated scenarios in Table 2 considering the respiratory information of 10 healthy subjects. Figure 10 shows the performance indices versus the threshold value employed in the developed automated crackle detector. Figure 10 top side shows the accuracy, sensitivity and specificity indices, while Figure 10 bottom side shows the receiver operating characteristic (ROC) curve. Selection of the threshold value employed in this study was based on these two results as follows. The optimum value for the pair sensibility-specificity was obtained from the ROC curve inflection and regarded as the lower limit (thr = 0.019). The upper limit for the threshold value was established as the maximum point for accuracy curve (thr = 0.029) where above this threshold the specificity achieves its maximum value and reaches a plateau while the sensitivity value reduced below 90%. Accordingly, the threshold value was set to the middle point between the lower and upper limits, i.e., thr = 0.024. Table 3 shows the results regarding the performance indices obtained with the selected threshold value (thr = 0.024) for all analyzes recordings with synthetic crackles inserted for each scenario with different signal-to-noise-ratios.

Regarding the crackle detection from recordings of nine patients diagnosed with DIP, the authors used the threshold value obtained during the simulated stage by the ROC curve, i.e., the threshold was 0.019. The results are summarized in Table 4 in terms of the number of crackles detected in each of the available recordings by the mobile app (via the automated crackle detection algorithm) and by the pneumologists (via the visual detection using TEWA criteria and audio playing of the signals). Due to the cumbersome manual detection of crackles by the pneumologists, just one respiratory cycle was analyzed from each recording. It is worth mentioning that in the case of the mobile app the complete acquisition and processing was performed in the developed mCORSA system. An example of the detection results from a DIP patient using the developed app is shown in Figure 6f, where it can be seen that the detected crackle sound resembles the morphology reported in the classical literature [3,9].

**Figure 11:**
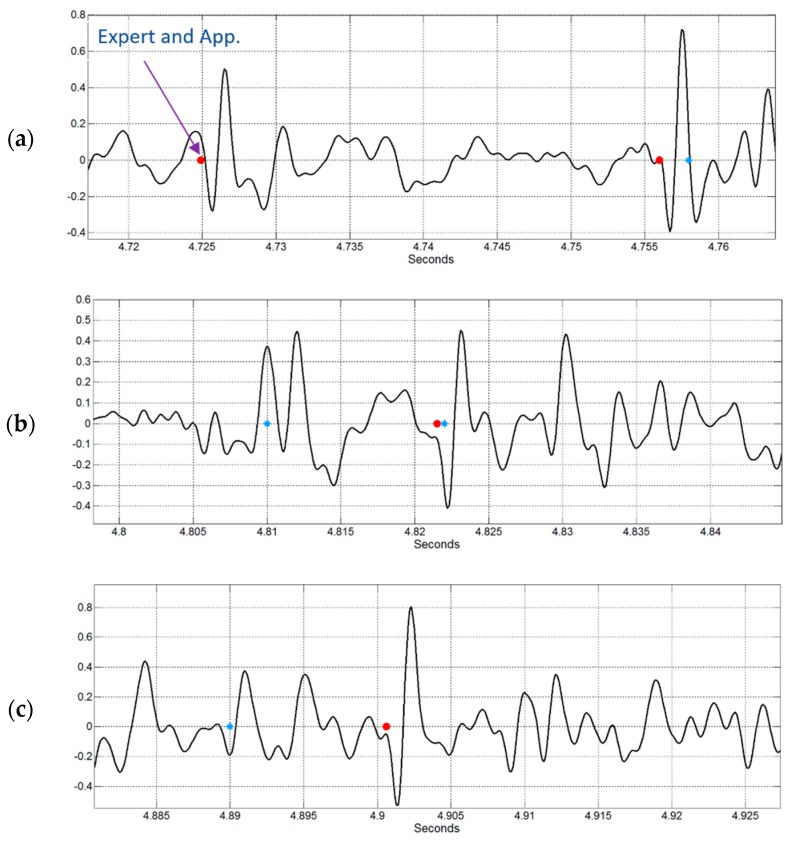
Examples of crackle detection performed by pneumologists (blue dots) and the developed smartphone application (red dots) in real acquired data from DIP patients. (**a**) Example of matching detection between pneumologists and the app. (**b**) Example of an extra detection provided by pneumologists but not by the app. (**c**) Example of an extra detection provided by pneumologists but not by the app and of a missing detection by pneumologists but detected by the app.

Regarding the performance indexes obtained for these real data, an average of 51% and 63% were found for the accuracy and specificity, respectively. Interestingly, a specificity of 100% was found in two cases where neither the pneumologist or the mobile app detected the presence of crackle sounds. It is worth mentioning that the detection of crackles that relies only on an auditory approach is difficult even for experts, as reported in the literature [2,43]. The task appears to be also challenging when using the TEWA criteria as illustrated by the examples shown in Figure 11, where the detected location marked by pneumologists and the app are represented by blue and red dots, respectively. Two examples of real crackles correctly detected by both pneumologists and the app are shown in Figure 11a where TEWA criteria correctly holds. Figure 11b shows an example where both the pneumologists and the app match the detection of a crackle sound (around 4.82 s) but the physician also indicates the presence of another crackle (around 4.81 s) which does not appear to resemble the classical morphology expected, while the smartphone does not detect it. Figure 11c shows an example when the app detects a crackle sound while the physician does not (around 4.91 s) although it follows the expected morphology. The additional crackle indicated by the physician (around 4.89 s) is another example of a case where doubts arise regarding the presence of a real crackle.

## 4. Discussion and Conclusions

In this paper, we proposed a smartphone-based CORSA system for the task of automated detection of adventitious discontinuous respiratory sounds, called crackles. In particular, we employed commercial Android devices and an acoustical sensor designed by our research group for the recording of respiratory sounds. A mobile app was implemented in Android Studio to govern the data acquisition, pre-processing, processing and display of results. The automated detection of crackles was performed by detecting abrupt changes in the coefficients of a time-varying autoregressive model of the recorded sounds. Real respiratory sounds from healthy and patients diagnosed with diffuse interstitial pneumonia were acquired using the developed mCORSA system.

The developed mCORSA system was designed to allow the physician to record respiratory sounds at the primary contact point which represents the first approach employed in the complicated and often long-lasting diagnosis of respiratory diseases. To this end, the core of the system is the mobile app designed with a friendly graphical user interface that allows one to introduce information from patients and to acquire respiratory sounds with the aid of a respiratory maneuver displayed in the device. The recorded sounds can be completely processed in the device and the detection results are provided in terms of the signal waveforms and as a summarized table for the respiratory phases. In addition, the app offers flexibility to load and analyze recordings obtained from another systems.

The automated algorithm for crackle detection was implemented in the app using Java programming language via an object-oriented approach. Because this language is not intended for numerical computation, a set of classes with their correspondent methods were implemented to perform the signal pre-processing and processing stages, including the digital filtering using FIR filters, direct and inverse FFT operations, as well as the RLS algorithm to estimate the TVAR coefficients of the recorded sounds. Implementation of these stages in the mobile device was compared to their counterpart in Matlab running in a personal computer, and no statistically significant differences were found (*p* > 0.99). The computation time and energy performance tests of the developed mobile app seems to show the feasibility of being used to record and analyze respiratory sound in an online scheme during several consecutive recordings performed directly in point-of-care settings. It is worth mentioning that the algorithms implemented in the smartphone device are subject to further improvement to reduce the computational time employed during the pre-processing stage, mainly the digital filtering scheme.

After testing a reliable estimation of the TVAR coefficients of the smartphone-recorded sounds, the performance of the automated crackle detector was tested using simulated scenarios found in clinical practice. Six different scenarios were created by randomly inserting synthetic crackles in basal respiratory sounds from healthy subjects with a variety of insertion timing along the respiratory cycles, signal-to-noise ratios, and crackle types. The threshold value employed in the crackle detection was selected using information from the ROC and accuracy curve. Regarding the detection of inserted fine crackles, it was performed, on average, with an accuracy ranging from 84.86% to 89.16%, a sensitivity ranging from 93.45% to 97.65%, and a specificity ranging from 99.82% to 99.84% for the studied scenarios. For the scenarios involving coarse crackles the average values were found to range from 57.95% to 85.18% for the accuracy, from 64.02% to 94.68% for the sensitivity, and from 99.83% to 99.85% for the specificity, respectively. For scenarios with combined fine and coarse crackles during the inspiration, the performance of the algorithm shows an average behavior between those previous two, with an average accuracy, sensitivity and specificity around 81%, 89%, and 99%, respectively. As can be noticed, the automated detection of crackle sounds is a more challenging task for coarse crackles than for fine crackles, perhaps due to their lower frequency content, compared to the one of fine crackles, which in turn becomes more masked by the frequency components of respiratory sounds with more power located at lower frequencies where it is also recognized that inspiration has a broader frequency band than the expiration. Consequently, the TVAR coefficients produced by the RLS does not change as much in comparison with the background as those associated with fine crackles, see Figure 5b. Other studies have also reported an increase in the failed detection of coarse crackles in comparison to fine crackles [43]. Accordingly, in the simulated scenario where fine crackles (higher frequency) were only inserted in the inspiration and coarse crackles (lower frequency) were only inserted in the expiration, the performance indexes increased with an accuracy, sensitivity and specificity around 91%, 95%, and 99%, respectively. Results from estimated locations of inserted crackles was found to be consistent for the different simulated scenarios and an average error distance ranging from 0.18 to 0.19 ms was found. The median value of the error distance for correctly detected crackles was found to be equal to 2 samples from the actual inserted location.

Regarding the crackle detection results from DIP patients, the obtained results show that the mobile app tends to underestimate the number of crackles present in the recordings in comparison with the pneumologist. Interestingly, in the recordings where the expert did not detect the presence of crackles using audiovisual labeling the proposed algorithm also reflects this absence or detect a minimum amount. Although the detection results obtained in real data seems to greatly diverge from the ones from simulated scenarios as accuracy reduced to around 52%, it is worth mentioning that the audiovisual detection is a time-demanding and cumbersome procedure while the mobile app performs this task faster and with repeatability from one analysis time to another. In addition, the detection of crackle sounds performed by expert physicians is not exempt of errors and it depends on their training and auscultation abilities. As illustrated in the Results section, pneumologists could provide extra detections and miss crackles based on their experience and limitations. Variability among physicians has been reported in the literature regarding detection of crackle sounds [43]. We consider that the detected locations obtained automatically by this and similar systems could help to validate the initial detection provided by the experts.

Although the results look promising, this study presents several limitations. First, the number of patients employed for validation is still limited and more real data is required to further evaluate the proposed system. Although the developed mCORSA system allows to record and analyze recordings easily and fast, its labeling by the pneumologist is not. Second, the performance of the developed system relies on the selection of the threshold detection value which was based on simulated scenarios. Although crackles were simulated accordingly to mathematical functions emulating real crackles found in clinical practice and with different signal-to-noise ratios and timing locations, the results from real data reflects that these functions would not capture all the dynamics of the underlying crackle generation. We consider that the development of a big database with real acquire respiratory sounds with adventitious sounds from patients will result beneficial to the improvement of this and similar detection algorithms, and that this data collection task would be greatly aided with this and similar mCORSA systems.

Current work involves the implementation of signal processing techniques that complement the information obtained from the waveform analysis by the physician. Future work will involve: (a) the development of a wireless version of the acoustical sensor that will enable data acquisition during a diversity of maneuvers involving conditions not restricted to stationary subjects, (b) improvement in the detection of coarse crackles by evaluating other techniques as independent component analysis and high-resolution time-frequency analysis, and (c) to add more microphones to count with spatial information. In addition, given the mobility characteristics of the system, it is being used to record a greater number of recordings in the clinical setting, directly at the bedside of the patients, which will be useful to develop more robust detection algorithms as well as to analyze them.

Finally, the authors emphasize that the developed mCORSA system was intended to provide an accessible tool to quantitatively analyze respiratory sounds while maintaining the desired properties of the auscultation like being non-invasive, ubiquitous, low-cost, and easy-to-use. In particular, the mobility characteristic of the system allows one to record respiratory sounds at the bedside of the patients without the need to move them, for example, to a specialized laboratory with an anechoic chamber. We consider that this and similar efforts will enable the acquisition of large samples of data with the benefits of a better understanding of pulmonary pathologies at early stages of the disease which in turn can help to deal with the underestimation of respiratory diseases among the general population that without the availability of mobile solutions found difficult to access, or access to late, to the specialized levels of the healthcare system.

## Figures and Tables

**Table 1:** Algorithm for automated detection of respiratory crackle sounds.

Step	Processing Stage
1.	Estimate the coefficients of the RLS-TVAR model of $s_{FN}\left[n\right]$.
2.	Obtain the derivate of each TVAR coefficient time series (CTS) to enhance abrupt changes due to crackles.
3.	Segment each derivative in 4 ms windows and compute the standard deviation in each window
4.	In each window, if the standard deviations of the four derivative CTS are above the thr value, then add the absolute values of the derivatives.
5.	In each window, obtain the maxima of the sum of the absolute values of derivatives
6.	In each window, if in the maximum point exist a slope change (positive to negative), then that point indicates the initial point of a respiratory crackle

**Table 2:** Simulated scenarios for performance evaluation of the proposed apps.

Scenario	Condition of Simulation
1	10 fine crackles inserted along each inspiratory phase
2	10 fine crackles inserted at the end of each inspiration
3	10 coarse crackles inserted at end of each inspiration
4	5 fine crackles and 5 coarse crackles inserted along each inspiration
5	10 coarse crackles inserted along each expiration
6	10 fine crackles inserted along each inspiration plus10 coarse inserted along each expiration

**Table 3:** Performance indices of the automated crackle detector implemented in the app for all simulated scenarios with inserted crackles to basal respiratory sounds from healthy subjects (*N* = 10).

Factor	Inserted Crackles	*Acc* (Unitless)	*Sen* (Unitless)	*Spec* (Unitless)	$\left|\left|\Delta t_{loc}\right|\right|\;(\mathrm{Ms})$
Scenario 1: Inspiratory fine crackles					
1.5	620	89.16 ± 4.46	97.65 ± 1.82	99.84 ± 0.12	0.19 ± 0.03
2.5	620	88.60 ± 4.29	97.45 ± 2.11	99.83 ± 0.12	0.18 ± 0.04
3.5	620	88.01 ± 3.60	97.11 ± 2.07	99.82 ± 0.11	0.18 ± 0.05
Scenario 2: Late inspiratory fine crackles					
1.5	620	86.77 ± 3.96	95.05 ± 2.31	99.84 ± 0.11	0.19 ± 0.03
2.5	620	85.73 ± 4.01	94.46 ± 1.87	99.82 ± 0.11	0.18 ± 0.04
3.5	620	84.86 ± 2.61	93.45 ± 2.37	99.83 ± 0.09	0.18 ± 0.05
Scenario 3: Late inspiratory coarse crackles					
1.5	620	74.76 ± 13.08	81.46 ± 12.72	99.84 ± 0.10	0.19 ± 0.03
2.5	620	82.49 ± 7.05	89.87 ± 4.22	99.84 ± 0.10	0.18 ± 0.04
3.5	620	83.75 ± 6.50	91.00 ± 3.96	99.85 ± 0.10	0.18 ± 0.04
Scenario 4: Inspiratory fine and coarse crackles					
1.5	620	79.70 ± 8.28	86.94 ± 6.64	99.84 ± 0.10	0.19 ± 0.03
2.5	620	83.03 ± 6.09	90.37 ± 3.78	99.85 ± 0.10	0.19 ± 0.04
3.5	620	83.10 ± 6.19	90.46 ± 4.07	99.85 ± 0.10	0.18 ± 0.04
Scenario 5: Expiratory coarse crackles					
1.5	530	57.95 ± 19.46	64.02 ± 19.55	99.83 ± 0.12	0.19 ± 0.03
2.5	530	81.50 ± 10.34	90.48 ± 7.68	99.83 ± 0.11	0.19 ± 0.04
3.5	530	85.18 ± 7.41	94.68 ± 4.00	99.83 ± 0.12	0.18 ± 0.04
Scenario 6: Inspiratory fine crackles plus expiratory coarse crackles					
1.5	1150	91.66 ± 3.14	96.16 ± 1.91	99.84 ± 0.09	0.18 ± 0.04
2.5	1150	91.37 ± 2.89	95.70 ± 1.73	99.85 ± 0.09	0.18 ± 0.04
3.5	1150	90.99 ± 3.95	95.54 ± 2.54	99.84 ± 0.09	0.18 ± 0.04
Data presented as mean ± standard deviation for all sound recordings					

**Table 4:** Crackle detection results in recordings from DIP patients (*N* = 9) using the automated detector implemented in the app and by a pneumologist.

Patient	Recording # 1		Recording # 2		Recording # 3	
	App	Physician	App	Physician	App	Physician
1	7	10	4	8	--	--
2	2	3	2	0	0	0
3	7	9	7	11	11	16
4	5	4	6	4	7	6
5	4	5	8	6	5	4
6	1	0	0	0	7	7
7	7	6	3	8	--	--
8	1	7	2	2	11	7
9	4	5	6	8	12	9
Total	38	49	38	47	53	49
